# An advisory program for first- and second-year medical students: the Weill Cornell experience

**DOI:** 10.3402/meo.v18i0.22684

**Published:** 2013-11-29

**Authors:** Lewis M. Drusin, Linda M. Gerber, Carlyle H. Miller, Carol L. Storey-Johnson, Bruce L. Ballard

**Affiliations:** 1 Departments of Public Health and Medicine and The Office of Academic Affairs, Weill Cornell Medical College, New York, NY, USA; 2 Departments of Public Health and Medicine, Weill Cornell Medical College, New York, NY, USA; 3 Department of Medicine and The Office of Student Affairs, Weill Cornell Medical College, New York, NY, USA; 4 Department of Medicine and The Office of Academic Affairs, Weill Cornell Medical College, New York, NY, USA; 5 Department of Psychiatry and The Office of Student Affairs, Weill Cornell Medical College, New York, NY, USA

**Keywords:** anonymous, questionnaire, satisfaction, academic, professional, research, career, evaluation

## Abstract

**Purpose:**

First-year students negotiate new professional culture with a certain amount of excitement and anxiety. There are different approaches for offering guidance. In this study, the authors present Weill Cornell Medical College's experience with an advising program for first- and second-year students.

**Methods:**

Fifty faculty advisors were each assigned 1–3 first-year students who they would follow for 2 years. The responsibilities were outlined to both faculty and students. The program was evaluated using an anonymous questionnaire.

**Results:**

For the two classes surveyed (2011 and 2012), most students met their advisors once. For both classes, the most frequently discussed issues were general adjustment to medical school, academic life, and the professional life of the advisor. Summer research and career opportunities were also discussed. Most students were satisfied with the advising program. Satisfaction increased with an increase in visits. Most students who did not meet their advisors established an advisor relationship on their own.

**Conclusions:**

An advising program was established at Weill Cornell Medical College that satisfied most of the students. It is important to evaluate its format regularly, from both student and advisor perspectives, in order to ensure its continued success.

## Introduction

First-year students starting in medical school negotiate a new professional culture with a certain amount of excitement and anxiety. The literature suggests that many medical schools use different formats with some degree of success. Examples of these formats include using focus groups to identify important parameters (1); allowing mentoring relationships to evolve through informal rather than assigned student–faculty contact (2); randomly matching groups of students to a faculty member who meets with a small group of students weekly (3); assigning an individual advisor to each student, with an attempt to match them using background information (4); dividing the student body into groups, each headed by a faculty member who directs a very structured program (5–7); establishing advisory colleges, with students equally distributed in them, and integrating this into wellness programs and personal development programs (8, 9); linking advisory functions with the responsibilities of following the advisee's academic progress and writing the first draft of the Dean's Letter (10); using an electronic journal to keep advisors up to date on their advisees’ progress (11); using alumni as career counselors for medical students (12); and assigning a faculty member to attend classes during the first and second years (13). There are advantages and disadvantages to each of these programs, but a single clear pathway to success has not emerged.

Over the years, Weill Cornell Medical College (WCMC) has offered several different components in an overall advisory system. For many years, we have had a very successful residency application advisory system in the third year that has resulted in excellent residency matches. The advisors in that system are specialty specific and work with students who have already decided on their individual choices of specialty. In cases involving academic difficulties, the Offices of Student Affairs and of Academic Affairs facilitate students receiving advice from course directors, which has been our most successful means of addressing any student's academic problems. Students who feel they are having any type of emotional problem utilize a Student Mental Health Service with full confidentiality. More informal opportunities for students to meet faculty include evening panels, sponsored by the Alumni Association, where alumni share their experiences in different specialties. Recently, we initiated a series of parties that are hosted each month by a different group of basic science or clinical departments. Also, first-year medical students are assigned to a second-year medical student to advise them on a smooth transition to medical school.

Students, however, often expressed a need for a general advisory system to start early in medical school that might assist them in later career decisions, impart the experiences of currently practicing physicians, and increase their awareness of what awaits them as they complete medical school and pursue specific specialties. Students also noted that there was no good way of obtaining a general faculty advisor unless the student took the initiative to seek out such an advisor.

In the late 1990s, as we revised our curriculum, we established a system of advisory ‘guilds’. Each had a cohort of faculty that met with an assigned group of students throughout medical school. These meetings were to provide greater interaction between students and faculty. Each group of faculty consisted of physicians from multiple medical specialty areas. Meetings could include formal presentations, such as panels, open forums, and small-group discussions. The advisory faculties were also available for students seeking individual advice. The group interactions were supposed to familiarize students with a number of faculty members in a non-classroom setting and would hence increase the level of comfort that students would feel in seeking advice. This initiative, however, was not successful for a number of reasons, including difficulties in arranging meeting times with large groups of faculty, irregular attendance at any meetings or forums because of patient care demands, and persistent problems with faculty needing more specific definitions of what kind of advisory role they were to have. Students often complained that the goals of the system were not well defined. The guild system approach did not improve our general advisory system.

In an attempt to improve our advisory system, and in recognition of newly articulated imperatives to assist students more proactively in developing their professional identities (14), we initiated a general advisory program for first- and second-year medical students at WCMC. We now present our current experience, and the evaluation of this advising program, as another alternative and to identify factors that would be useful to incorporate in future adjustments to our advising system.

## Methods

The associate dean for student affairs and the program director identified approximately 50 busy, well-liked, and successful faculties who knew the medical college well and were interested in spending time with students. Their advisory role was outlined in a standard telephone interview. The advisors would make initial telephone or e-mail contact with 1–3 first-year medical students who they would advise for 2 years. They were asked to provide reassurance as the students became accustomed to medical school, to facilitate the students’ exploration of opportunities and introduce them to possible mentors as their interests evolved, and to be their advocates if problems developed. They also would help the students understand their new professional responsibilities. To facilitate the development of a one-to-one relationship, the advisors were reimbursed for taking their advisees to lunch individually as a first meeting.

During orientation in September of the first year, the program responsibilities of the advisors were outlined for the students, and they were told that they would each receive contact information for their randomly assigned, generic, faculty advisor.

Early in October, letters were sent to advisors and students outlining the program and providing contact information (telephone numbers and e-mails). Students were told to contact their advisors if they did not hear from them within 2 weeks. If they were still unable to set up a meeting, they were asked to notify the program director, who would facilitate the meeting.

In February, all students received an e-mail from the program director with copies to the advisors to suggest that it was time to start planning their summer experiences. In June, the associate dean and the program director asked the first-year class to complete an anonymous questionnaire to evaluate the advising program.

Survey data were collected during the first year for the Class of 2011 and were compared with similar data collected for the Class of 2012. The questionnaire was distributed to the students at the end of a lecture. Students were asked to complete it before they left the room. We performed all analyses using SPSS version 15.0 (SPSS Inc., Chicago, IL, USA).

The study was approved by the Weill Cornell Medical College Institutional Review Board.

## Results

Seventy-nine students from the Class of 2011 (*N*=103) and 53 students from the Class of 2012 (*N*=103) completed the questionnaire. For the Class of 2011, 83.5% (66/79) met with advisors (Table 1). However, 73.6% (39/53) of the Class of 2012 met with their advisors. The majority of the class met with their advisors at least once: 69.7% for the Class of 2011 and 71.8% for the Class of 2012. For both classes, the issues most frequently discussed were general adjustment to medical school, academic life, and the professional life of the advisor (Table 2). The majority of students in both classes were satisfied with these discussions. Summer research and career opportunities were discussed less frequently, although students were encouraged to discuss these topics, and the level of satisfaction was slightly higher for summer research and career opportunities for the Class of 2011. The big picture in medicine, although an ill-defined category, was more frequently discussed by the Class of 2012 and at a higher level of satisfaction than the Class of 2011. For both classes, the subject least discussed was the student's personal life.

**Table 1:** Distribution of students who met with their advisor, by class

	Yes (%)	No (%)	Total (*N*)
Class of 2011	83.5	16.5	79
Class of 2012	73.6	26.4	53
Total	79.5	20.5	132

**Table 2:** Topics discussed between advisors and students, by class

	Not discussed (%)	Not helpful (%)	Neutral (%)	Somewhat helpful or helpful (%)	Total (*N*)
General adjustment to medical school					
Class of 2011	4.8	3.2	21.0	71.0	62
Class of 2012	0	10.3	28.2	61.5	39
Total	3.0	5.9	23.8	67.3	101
Your academic life					
Class of 2011	6.2	1.5	32.3	60.0	65
Class of 2012	0	10.3	20.5	69.2	39
Total	3.8	4.8	27.9	63.5	104
Your personal life					
Class of 2011	35.0	1.7	28.3	35.0	60
Class of 2012	28.2	12.8	30.8	28.2	39
Total	32.3	6.1	29.3	32.3	99
Summer research opportunities					
Class of 2011	20.6	7.9	20.6	50.8	63
Class of 2012	12.8	17.9	28.2	41.0	39
Total	17.6	11.8	23.5	47.1	102
General research opportunities					
Class of 2011	28.6	6.3	22.2	42.9	63
Class of 2012	13.2	21.1	21.1	44.7	38
Total	22.8	11.9	21.8	43.6	101
Career opportunities					
Class of 2011	22.2	3.3	22.2	52.4	63
Class of 2012	10.3	12.8	25.6	51.3	39
Total	17.6	6.9	23.5	52.0	102
Professional life of the advisor					
Class of 2011	4.7	4.7	15.6	75.0	64
Class of 2012	0	7.7	23.1	69.2	39
Total	2.9	5.8	18.4	72.8	103
‘Big-picture issues’ in the field of medicine					
Class of 2011	34.4	1.6	18.0	45.9	61
Class of 2012	10.5	10.5	23.7	55.3	38
Total	25.3	5.1	20.2	49.5	99

Overall satisfaction was highest for the Class of 2011; it was 72.7%, compared to 56.4% for the Class of 2012 (Table 3). For both classes, the majority of students met with their advisors once (Table 3). For the small number of students in both classes who saw their advisors more than once, satisfaction increased with the increase in meetings.

**Table 3:** Ratings of overall satisfaction with advisor, and times met with advisor, by class

	Somewhat or very dissatisfied (%)	Neutral (%)	Somewhat or very satisfied (%)	Total (*N*)
Overall satisfaction with advisor				
Class of 2011	7.6	19.7	72.7	66
Class of 2012	15.4	28.2	56.4	39
Total	10.5	22.9	66.7	105
Times met with advisor				
Class of 2011				
1	10.9	23.9	65.2	46
2	0	25.0	75.0	8
≥ 3	0	0	100.0	12
Total	7.6	19.7	72.7	66
Class 2012				
1	14.3	35.7	50.0	28
2	40.0	0	60.0	5
≥ 3	0	16.7	83.3	6
Total	15.4	28.2	56.4	39

Unfortunately, the majority of students who did not meet with their advisors (91.7%, or 11/12, for the Class of 2011; and 76.9%, or 10/13, for the Class of 2012) failed to notify either the associate dean for student affairs or the program director. A small number of these students did acknowledge that their advisors had tried to contact them: 30.8% (4/13) in the Class of 2011 and 20.0% (3/15) in the Class of 2012 (Table 4). Also, a few students attempted unsuccessfully to connect with their advisors: 38.5% (5/13) in the Class of 2011 and 56.3% (9/16) in the Class of 2012. Only one student from the Class of 2012 admitted not choosing to have an advisor. Of all the students who did not meet their advisors, 84.6% (11/13) from the Class of 2011 and 21.4% (3/14) from the Class of 2012 were able to establish an advisor relationship with another faculty member on their own.

**Table 4:** Experiences of students who never met their advisors, by class

	Advisor attempted to contact you			Attempted to contact your advisor			Chose not to have an advisor			Chose an advising relationship with a different faculty member		
				
Class	Yes: *N* (%)	No: *N* (%)	Total	Yes: *N* (%)	No: *N* (%)	Total	Yes: *N* (%)	No: *N* (%)	Total	Yes: *N* (%)	No: *N* (%)	Total
2011	4 (30.8)	9 (69.2)	13	5 (38.5)	8 (61.5)	13	0 (0)	13 (100)	13	11 (84.6)	2 (15.4)	13
2012	3 (20.0)	12 (80.0)	15	9 (56.3)	7 (43.8)	16	1 (5.9)	16 (94.1)	17	3 (21.4)	11 (78.6)	14
Total	7 (25.0)	21 (75.0)	28	14 (48.3)	15 (57.7)	29	1 (3.3)	29 (96.7)	30	14 (51.9)	13 (48.1)	27

Four comments recurred frequently in the questionnaires. Both classes requested being matched to an advisor based on interest: 11.4% (9/79) for the Class of 2011 and 22.6% (12/53) for the Class of 2012. Both classes requested more frequent meetings between advisors and advisees: 10.1% (8/79) for the Class of 2011 and 11.3% (6/53) for the Class of 2012. Although 10.1% of the Class of 2011 requested scheduling meetings so that students do not intrude on faculty, the Class of 2012 did not comment on this. The students also wanted more contact between advisors and advisees: 7.6% (6/79) for the Class of 2011 and 5.7% (3/53) for the Class of 2012.

## Discussion

The published reports cited in this article discuss a continuum between mentoring and advising, illustrating many different formats that can achieve success. Each of them has a slightly different focus. The 50 first- and second-year advisors in our early advising program are male, female, Asian, Hispanic, African American, and Caucasian. They come from various clinical and research backgrounds and vary in academic rank. Gender, race, and academic discipline are not factors in their selection. They are chosen because they have a strong history of involvement with students, an in-depth knowledge of the medical center, and a willingness to participate. Although students from both classes have requested that advisors and advisees be matched by interest, it is not possible to guarantee that this can be done for more than 100 students. In addition, student interests change over time. Matching advisors by interest could create an awkward situation if a student's interest changes. The student might feel uncomfortable informing his or her advisor, and the advisor might not be willing to accept the student's loss of interest in his or her field. The generic advisors, on the other hand, are prepared to accommodate students’ evolving curiosity and identify the right mentor to facilitate their professional development once their interests have solidified.

The students were in favor of more frequent contact and meetings with their advisors. They requested scheduling meetings so that students did not intrude on the faculty. The data suggest an association between frequency of meetings and satisfaction with the program. Faculty chosen as advisors tended to be busy people who were nationally and internationally known in their fields, so that they would be excellent role models. Anecdotally, some students were reluctant about contacting them because they thought they were intruding in their busy, professional life. Some of the advisors thought that students who did not contact them were disinterested in the advising relationship. It seems important to foster a one-to-one relationship so that students having problems will feel comfortable talking with their advisors.

The associate dean and the program director were available to facilitate contact between advisors and advisees when this was difficult. It was surprising how few students took advantage of this. However, it was encouraging to find out that the majority of students who were unable to make contact with their assigned faculty advisor were able to establish an advising relationship with a faculty member of their own choosing.

One limitation in our study could be the sample size. We administered the questionnaire to both classes at the end of a lecture and asked them to complete it before they left the room. Although the demographic characteristics of the classes were similar, the number of responses from the Class of 2012 (53) was considerably smaller than the number from the Class of 2011 (79). The smaller sample size might in some part account for the different results in the two classes. It was not possible to evaluate the non-responders in this anonymous study. In spite of the sample size, we were able to gain insight into additional factors that might be incorporated into a more successful advisory program.

There has been very little turnover in the cohort of 50 advisors. The faculty who have left the program did so because they left the medical center. The program director speaks to all of the advisors just before the start of the academic year to remind them of their obligations and confirm their commitment.

An important strength of our advising system is the annual review, which includes the anonymous questionnaire as well as both focus-group discussions with the medical students and feedback via a structured interview with the advisors. This allows us to continuously monitor the program and make appropriate adjustments as needed. For example, in response to the annual review, we developed a list of resources for first-year advisors and five suggested topics for discussion. These include transition to medical school, stress management, wellness, professionalism, and summer opportunities. This feedback will allow us to put in place an enhanced structured advising program that will include an evaluative component.

In accordance with emerging views and recommendations that medical schools pay more attention to facilitating the professional development of medical students, it is a good time to rethink the many models that have been used thus far and improve them. We believe that early advising, beginning in year one, is an important feature of successful programs.

Differences in satisfaction responses between medical student classes in areas such as career, summer research, and the ‘big picture of medicine’ suggest that a developmentally appropriate, scripted advising system, in which discussions are tailored to the experiential level of the student, may have value. Traditionally, attendance at early advising systems has not been made mandatory in the same way that attendance at other required course components has, and this is reflected in the variability of the data showing if and how often students connected with advisors. Interestingly, the high number of students who did not connect with their assigned advisors but who found advisors on their own suggests that there is a need for advising by all students in one way or another. The combination of the perceived need for advising by students, the potential value of developmentally appropriate and regularly scheduled opportunities for self-reflection, and addressing aspects of the hidden curriculum (15) that are encountered in the clinical arena, along with the development of a longitudinal relationship with one faculty advisor who can assist the student in accessing various institutional resources over all of the years of the student's educational program makes the development of a mandatory longitudinal scripted advising system, starting at year one, a very attractive model to pursue to help students develop their professional identity. This individual longitudinal experience with an advisor could be combined easily with structured group meetings as needed. We are in the early stages of developing such a program, and we hope to report on its successes and challenges in the future.

## Approval

The protocol has been approved by the Weill Cornell Medical College Institutional Review Board.
